# The Role of Artificial Lakes Located in Forests in the Context of Small Retention, Biodiversity and Climatic Changes—Evidence From Southern Poland

**DOI:** 10.1002/ece3.70775

**Published:** 2025-01-21

**Authors:** Rafał Starzak, Anna Cieplok, Robert Czerniawski, Aneta Spyra

**Affiliations:** ^1^ Department of Ecological Engineering and Forest Hydrology University of Agriculture in Krakow Kraków Poland; ^2^ Institute of Biology, Biotechnology and Environmental Protection, Faculty of Natural Sciences University of Silesia Katowice Poland; ^3^ Department of Hydrobiology, Institute of Biology University of Szczecin Szczecin Poland

**Keywords:** biological indicators, diversity, drought management, forests, phytolittoral assessment, water retention

## Abstract

Drought has an effect on hydrologic conditions and water quality under climate change. Small water retention in forests is one of the priority investment programs implemented in recent years, supported by the European Union. This study aimed to assess the ecological conditions of forest lakes using macrophytes and benthos organisms diversity as an ecological indicator of ecosystem conditions under climatic changes. The study was carried out in forest artificial lakes serving as surface water retention in the context of biodiversity in climatic changes and its role in the retention of water. Despite systematic maintenance activities, a long period of lake existence significantly determines the natural biological processes occurring in lakes and riparian habitats. The analysis showed low values of salinity indicators and the concentration of nitrogen and phosphorus. The pH ranged from 6.2 to 7.6; showing slightly acidic conditions or within the limits of neutral. The model of plant associations showed the occurrence of 24 species of plants within nine plant assemblages in the *Phragmitetea* and *Potametea* classes of associations (Biocenotic index 1.007–1.692). Despite human activities, lake condition, as assessed by the ESMI index or the biocenotic diversity indices, is good (0.416–0.648). Climate change, expressed by an increase in the frequency of dry years, creates a situation of changes in filling lakes with water, which, taking into account their small depth, results in dynamically changing conditions for the development of phytolittoral. Along with the phytolittoral changes, benthos communities change, their density and the number of taxa also fluctuate. It should be assumed that with ongoing climate change, these phenomena will probably intensify, which will lead to changes in entire ecosystems at plant and animal levels.

## Graphical abstract

The model of plant associations showed the occurrence of 24 species of plants within nine plant assemblages in the Phragmitetea and Potametea classes of associations. Despite human activities, lake condition, as assessed by the ESMI index or the biocenotic diversity indices, is good.

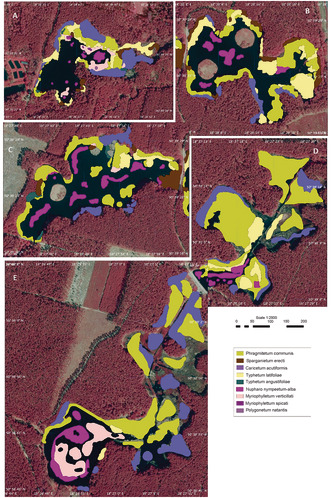

## 1 Introduction

With ongoing climate change, entire areas are being altered because drought affects an increasing number of land and aquatic ecosystems. Drying out of the aquatic environments has an influence on hydrologic conditions and water quality under climate change. It is predicted that drought, as a consequence of atmospheric warming and precipitation shifts, will affect global hydrology and water quality, resulting in a considerable challenge to water availability for society, the environment, and ecosystems (Qiu, Shen, and Xie 2023). This also influences water balance and quality across global land regions through changes in hydrological components and pollution, which threatens the water resources of ecosystems (Gøtske and Victoria 2021; Pokhrel et al. 2021). Climate change affects modifications in tree stands and groundwater quality but also affects aquatic environments located in forest areas (Lindner et al. 2010; Dao et al. 2023; Girona et al. 2023).

Forests are not adapted to changes; therefore, changes in tree stands are predicted to be wide because of changing climate variables and increased variability with greater risk of extreme weather events, such as prolonged drought, storms, and floods. In northern and western Europe, the increasing atmospheric CO_2_ content and warmer temperatures are expected to result in positive effects on forest growth and wood production, at least in the short‐medium term. On the other hand, increasing drought and disturbance risks will cause adverse effects (Lindner et al. 2010, 2014). According to the Report on the condition of forests in Poland in the period 2020–2022, drought was the main disaster phenomenon in a national range. In forest stands at age over 20 years, which are managed by the National Forests, the area from 29.3 to 62.4 thousand ha of trees (0.4%–0.9% of forests managed by the National Forests) were recorded as significantly damaged by the disturbances in water conditions. Symptoms of weakening or damage to forest stands caused by disturbance of water conditions, mainly drought, were recorded in 173 forest districts (29,300 ha, 0,41%) (PGL LP 2020; PGL Państwowe Gospodarstwo Leśne Lasy Państwowe 2021, 2022). In previous years, the situation was similar; in 2020, it was 62,400 ha (0,9%) in 253 forest districts, and in 2021, it was 35,600 ha (0,5%) in 168 forest districts.

The long‐term and constant lack of water weakens the natural defenses of many organisms (Cooke et al. 2022; de Melo et al. 2022; Jitariu et al. 2022). They become more susceptible to disease, pests, pathogens, and extreme weather events. The risk of fires in forests increases with persistently high temperatures and long‐term lack of rainfall. The dominant conifer species, dried litter, and the presence of deadwood favor the rapid spread of fire (Hueso‐González, Martínez‐Murillo, and Ruiz‐Sinoga 2018; Cerdà et al. 2022; Kupka et al. 2022). During periods of drought, the herbaceous vegetation of most species dries out, which results in the depletion of the food base of other organisms. Living and breeding conditions for many water‐related species deteriorate, and in extreme cases, they can be at risk of extinction. One of the effects of water scarcity, which is extremely harmful to nature, is the reduction of biodiversity (Harvey et al. 2023; Zhang et al. 2023). Aquatic invertebrates also respond to climatic changes, temperature growth, and drying out of water systems. The density of a common group of invertebrates, such as Chironomidae larvae, increased during the drought years, but the richness and abundance of other groups, such as, for example, mayfly, stonefly, and caddisfly (EPT) taxa declined during the late drought (Herbst et al. 2019). Therefore, in consequence, the species diversity decreases dramatically, especially in extreme drought events, which can lead to local biotic homogenization in water bodies (Bertoncin et al. 2019).

Small water retention in forest complexes is one of the priority investment programs implemented in recent years by the National Forests State Forest Holding (Centre for Coordination of Environmental Projects). These activities are supported by European Union funding. The effects of activities in the small retention are usually expressed in the amount of water retained in aquatic reservoirs or qualitative landscape changes (Mioduszewski 2014). The habitat‐forming and biocenotic role of reservoirs is treated a priori and there are very few scientific data on this issue. It seems that one of the reasons for this is the lack of methodology for assessing the impact of reservoirs on biodiversity, especially in the absence of environmental monitoring prior to the implementation of the investment.

The presence of macrophytes has a positive effect on the condition of the ecological lake and provides competition for phytoplankton in the uptake of nutrients (Ciecierska and Kolada 2014; Pełechaty and Pronin 2015; Pietruczuk, Dajewski, and Czarnecki 2020). This study aimed to assess the ecological conditions of forest reservoirs using macrophytes in the context of the ecological conditions of artificial lakes and the assessment of benthos organisms diversity as an ecological indicator of ecosystem conditions under climatic changes. Aquatic macrophytes act as important bioindicators of environmental conditions and long‐term ecological changes in water quality (Solimini et al. 2006). In view of the important function of macrophytes in aquatic ecosystems, understanding and quantifying the environmental factors that influence the distribution patterns of macrophytes are indispensable for integrated management practices of these ecosystems. The manifold role of aquatic plants in freshwater systems is closely linked to their distribution, which in turn depends on a variety of parameters. This study is important in order to show the role of artificial lakes located in forests in the context of small retention, biodiversity, and climatic changes because of changing environmental stability and intermittence of aquatic water bodies.

## 2 Material and Methods

### 2.1 Study Area

This research was carried out in the forest complex of small, artificial lakes, which are objects of surface water retention. The first lakes in this complex originated 200 years ago (probably about 1820). This facility is located in the eastern part of the Opole Plain in the catchment area of the Mała Panew River (the right tributary of the Oder River). The studied complex of lakes is located in a wide area of forest complex Stobrawsko‐Turawskie Forests, which is not diverse in the species and habitats context. The advantage of this locality is the long period of existence of lakes, which, despite systematic maintenance of human activities, significantly determines the natural biological processes occurring in the forest lakes and in riparian habitats.

It is an area of bottom moraine elevated from 150 to 200 m above sea level with characteristic old glacial dunes (Galon 1972; Starkel 1999), and is located in the valley of the Smolina River (Odra River basin). The main source of water supply is the Lublinica River, from which water is supplied to the reservoirs by a canal. Pleistocene River sands of accumulation terraces of the Northern Polish glaciation occur in the Mała Panew valley (Kotlicki 1971; Przybylska 2009). Quaternary sediments form a cover with a thickness of 15–25 m, and the water within this aquifer is free at a depth of 0.8–4.0 m (1.5 m amplitude of fluctuations in the annual cycle). In the study area, rusty soils have formed (WRB07: Brunic Arenosols; Soil Taxonomy: Inceptisoils [Typic Udipsamments]) and rusty podzolic soils (WRB07: Albic Brunic Arenosols; Soil Taxonomy: Inceptisoils [Spodic Udipsamments]) and proper podzolic soils (WRB07: Haplic Podzols; Soil Taxonomy: Spodosoils [Haplorthods]). Locally in watercourse valleys, gley soils (WRB07: Haplic Gleysols; Soil Taxonomy: Entisoils [Endoaquents]) also occur. Rusty soils are acidic, with a pH range from 3.5 to 5.0, and with low saturation of the sorption complex with alkaline cations (< 30%). The C:N ratio is in the range of 15–20:1. Podzolic soils are strongly acidified (pH 3.0–4.5), and the degree of saturation of the sorption complex with alkaline cations is low (less than 20%), and the C:N ratio is 20–30:1 (Skłodowski 2014). The tree stand surrounding the studied forest lakes is a compact complex of coniferous forests, growing on poor habitats of fresh coniferous forest and fresh mixed coniferous forest. The dominant species in the tree stand is pine ( *Pinus sylvestris* L.) occupying about 93% of the area. The other species of trees are silver birch ( *Betula pendula* Roth) and Norway spruce ( *Picea abies* (L.) H. Karst). In the shrub layer, *Padus avium* (Mill.) occurs. Potential natural vegetation in the study area is continental mixed pine‐oak forests (*Querco‐Pinetum*) (Matuszkiewicz et al. 1995; Matuszkiewicz 2008).

**FIGURE 1:**
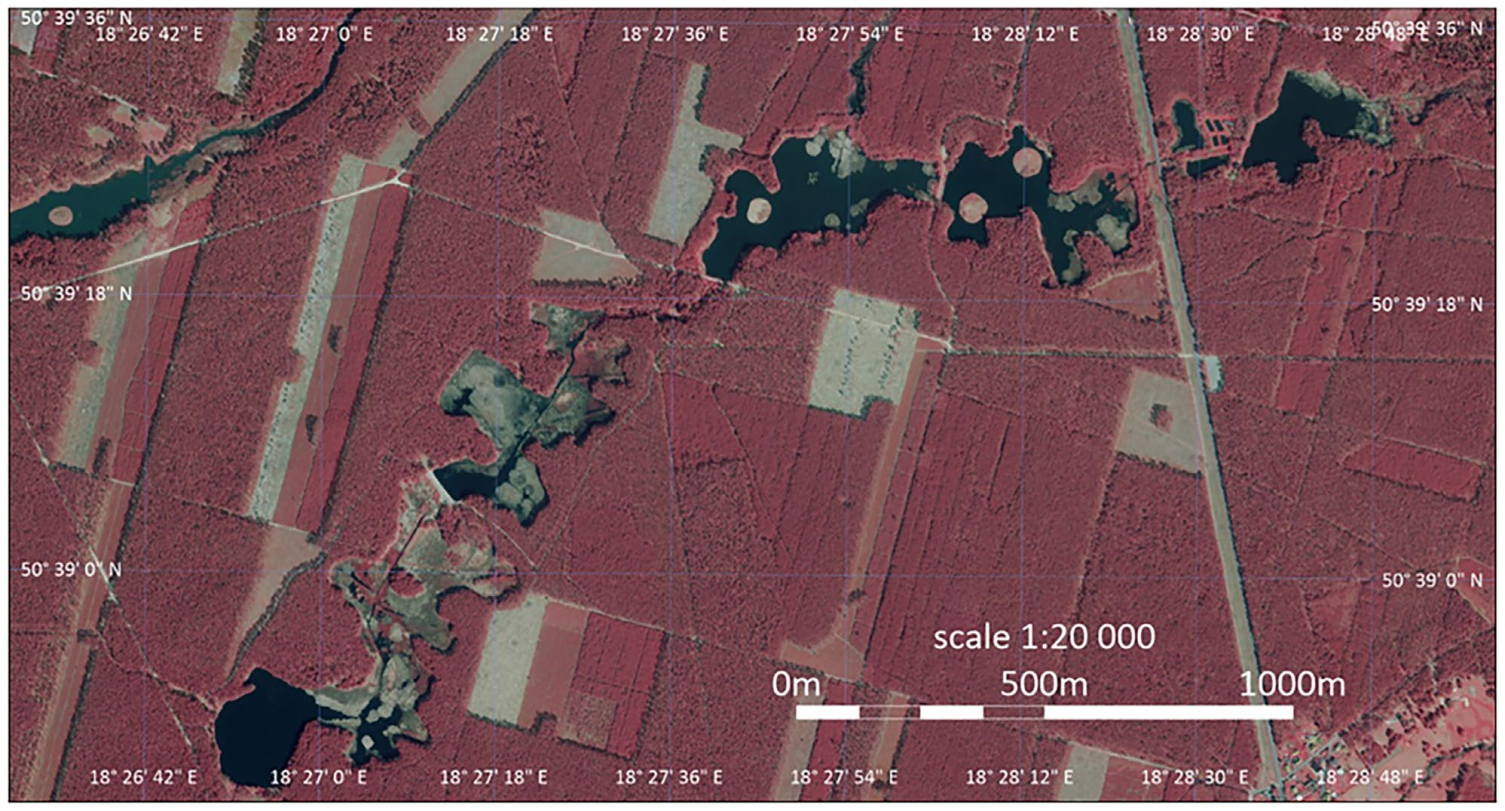
The map constructed for the entire studied complex of artificial forest lakes. Number of lakes–5.

The climate characteristics were prepared on the basis of data provided by the Institute of Meteorology and Water Management—National Research Institute. Data from the period 1991–2014 from the meteorological station in Stare Olesno (N 50.90 E 18.36), located 30 km in a straight line from the lakes studied, were used. The average long‐term annual temperature is 8.3°C, and the average long‐term temperature of the growing season (April–September) is 14.7°C. The average rainfall in the period 1991–2014 is 669 mm for the annual period and 410 mm for the growing season (April–September) (Figure 1). The highest average monthly temperature of 18.7°C is recorded in July, and the lowest −1.6°C in January. The highest precipitation is in the summer months. The highest multiannual average monthly precipitation is 95 mm in July, and the lowest is 35 mm in February. The balance of the water in the atmosphere calculated using the Thornthwaite method (Thornthwaite and Mather 1955, 1957; Westenbroek et al. 2010) is moderately positive per year—the excess of precipitation is 43 mm. In a monthly context, the amount of precipitation exceeds the capacity of the atmosphere to absorb water vapor for about 6 months (October to March). About 60% of the precipitation falls from May to September. Similarly, the highest values of potential evapotranspiration are recorded in the months of the growing season, as much as about 75% of the annual value. The characteristic features of the studied lakes are presented in Table 1.

**TABLE 1:** The characteristic and morphometric features of forest lakes studied. 1–5—number of lake MLT—minimum number of transects.

	1	2	3	4	5
Locality of sampling site	The west part of lake	The north part of lake	The north part of lake	The south‐western part of lake	The south part of lake
Geographic coordinates of sampling point	50.658104° 18.476545°	50.657669° 18.468885°	50.657616° 18.466591°	50.651901° 18.453382°	50.646828° 18.447107°
Exposition to sunlight	Sun‐exposed	Sun‐exposed	Partially shaded	Sun‐exposed	
Bottom sediments	Muddy with a layer of detritus	Sandy‐muddy	Muddy with a large amount of plant remains	Sandy‐muddy with a thin layer of detritus	Sandy‐muddy with plant remains and submerged macrophytes
Water depth in sampling point [cm]	120	30–60	10–40	30–60	30
Characteristic of shoreline	Steep shore	The entire edge of the reservoir reinforced with concrete fascines	Wide strips of reeds entering the central part of the pond	The west part of the pond reinforced with concrete fascines	The southern shore covered with submerged vegetation
Lake area [ha]	3.92	7.93	8.17	8.99	13.66
Water elevation [m n.p.m.]	217	216	215	213	211.8
Average depth of pond [m]	1.1	1.2	1.2	1.2	1.85
Lake circumference [m]	1322	1713	1686	2298	3028
Water volume [m^3^]	38.940	86.040	90.600	95.760	231.990
MLT	4	3	3	4	5

The studied complex of small lakes is of anthropogenic origin. The first mention of their existence in this area is from 1880 (Lublinitz 1880). Currently, lakes are owned by the State Forests National Forestry—Zawadzkie Forestry (Regional Directorate of State Forests in Katowice). In the years 2006–2007, the construction of damming structures between the lakes and permanent overflows of the water was carried out. The recommended maintenance procedures in addition to the maintenance and conservation of hydrotechnical equipment, include, among others, mowing dike slopes or removing vegetation in the forest lakes.

### 2.2 Reservoir Morphometry

The current range of the water surface in the small artificial lakes was determined based on GPS measurements using the Garmin 64 receiver. The size of the plant communities in the ponds was measured from a pontoon using a measuring device. Direct measurements from the pontoon included depth, which was determined by probing the ponds with a weighted tape. Approximately 30 depth soundings were made for each pond. The perimeter of the ponds and their capacity were calculated from the field measurements using the Surfer computer program (Golden Software). The locations of the bottom sediment collection points were measured and tabulated using a Garmin 64 GPS receiver (Table 1).

### 2.3 ESMI Method (Ecological State Macrophyte Index)

The study of macrophytes was carried out in July–August 2021 at the peak of the growing season. Vegetation studies were carried out using the sigma‐photo. The area of the image is usually the area of the transect. The minimum number of transects MLT depends on the size of the lake and the degree of development of the shoreline. For lakes smaller than 20 ha, it is calculated using the Jansen formula (Jensén 1977; Keskitalo and Salonen 1994):

$$\mathrm{MLT}=\frac{L}{\sqrt{\mathrm{\pi P}}}$$

where L—the shoreline length in km, P—the surface of lake in km^2^.

Since the calculated number of transects (Table 1) was lower than the minimum number of 6 transects provided for in the method, it was assumed that the research would cover entire lakes and measure the areas of each identified community. The study of vegetation in the lakes was carried out visually, by observation from a watercraft, determining the limits of its occurrence using a GPS device and direct measurements with a measuring tape. The small depth of the reservoirs (max 2 m) and high water transparency allowed using the same measurement techniques in the case of submerged vegetation. The term “community” is understood as a cluster of plants of a given species, covering an area of ≥ 1 m^2^ and with a coverage density of at least one‐quarter of the area.

Ecological State Macrophyte Index—ESMI was calculated using its version for shallow lakes (Ciecierska et al. 2006):

$$\text{ESMI}=1-\exp\left(-\frac{H}{H_{\max}}∙Z∙\exp\left(\frac{N}{P}\right)\right)$$

where *H*—biocenotic diversity index, Hmax—maximal biocenotic diversity *Z*—occupation index, *N*—the total area of the phytolittoral (100%), *P*—lake surface (km^2^).

The biocenotic diversity index (H) is the modification of the Shannon index (Shannon and Weaver 1949), where the quantitative feature is the area of individual communities, according to the formula:

$$H=-\sum \frac{n_{i}}{N}\times \ln\frac{n_{i}}{N}$$

where *n*_i_—the area of patches of a given plant community, expressed as a percentage of the total area of the phytolittoral.

Theoretically possible maximum biocenotic (phytocoenotic) diversity (Hmax), was calculated from the formula:

$$H_{\max}=\ln S$$

where S—number of communities forming a phytolittoral.

The ratio of the last two indices (H and Hmax) is understood as a measure of the structural simplification of vegetation under the influence of anthropopressure and is known as the evenness index (Pielou 1966):

$$J=\frac{H}{H_{\max}}$$

As a measure of macrophyte abundance, the occupation index (Z) was adopted, which expresses the ratio of the area actually occupied by macrophytes (phytolittoral area) to the area potentially available for plants, for example, the littoral area limited by the 2.5 m isobath (area of the lake where the water is shall lower than 2.5 m). In the case of the small lakes in this study, it was the total area of the reservoir. The settlement rate is, therefore, calculated according to the formula:

$$Z=\frac{N}{P_{\text{isob}\;2.5}}$$

where *Z*—occupation index; *N*—total area of the phytolittoral (ha); *P*—the surface of the lake isob. 2,5—area limited by isobath 2,5 (from the morphometric card) (ha).

The patches of actual vegetation from 2021 are presented in view of coverage from March 20, 2019, which is available in the form of satellite photography. Orthophoto maps (M‐34‐49‐B‐b‐2‐1 and M‐34‐49‐B‐b‐2‐2) were used in ASCII format with a spatial resolution of 1.0 m and an average height error of 0.15 m.

### 2.4 Sampling Procedure of Hydrobiological Studies

Studies were carried out in the five forest lakes. The hydrobiological sampling includes taking samples three times during the study period in each lake: in spring (May), summer (August), and autumn (November). Sampling points were selected considering accessibility to the shoreline, safety during sampling, depth at the sampling point, and diversity of microhabitats.

We collected benthic invertebrate samples using the quantitative method from an area of 50 cm^2^, using a bottom scraper from bottom sediments to a depth of 25 cm. In total 45 samples were taken (water, bottom sediments, and invertebrates). The collected biological material was put into plastic containers and transported to the hydrobiological laboratory at the Faculty of Natural Sciences, the Institute of Biology (Biotechnology and Environmental Protection, the University of Silesia, Katowice) for further analysis. The material was rinsed on a sieve with a mesh size of 0.4 mm. From each sample, benthic organisms were selected and preserved in 80% ethanol. Benthic invertebrates were identified on the basis of morphological features based on specialist identification keys: Pawłowski (1936), Rozkosny (1980), Tończyk, Bernard, and Buczyński (2000), and Czachorowski and Pietrzak (2003) using a stereoscopic microscope OLYMPUS (SZX16) with OLYMPUS and Cell Sens Standard 1.4 (Build 8583, Olympus Camera Software Ver. for Microsoft Windows NT 6.1). Most organisms were classified to family level; Megaloptera larvae, leeches, and bugs were identified to species rank. The density of the collected organisms was calculated per 1 m^2^ of the bottom surface.

Along with the benthos sampling, we sampled also the water to analyze the physical and chemical properties. Directly during the sampling, parameters such as temperature, pH, conductivity (EC), the content of dissolved solids (TDS), and the content of dissolved oxygen (DOX) were measured using HI 9811–5 pH/EC/TDS/°C portable meter (Hanna Instruments, Woonsocket, RI, USA). Other parameters were analyzed in the laboratory. We measured the concentration of chlorides, calcium, iron, potassium, nitrates, nitrites, sulfates, phosphates and ammonia, alkalinity, and hardness of the water in the samples using a titrimetric determination method and reagents from Merck and Hanna Instruments (Darmstadt, Germany, Woonsocket, RI, USA).

### 2.5 Organic Matter in the Bottom Sediments Method

We collected the samples of bottom sediments to analyze the organic matter content in each lake. After collecting the bottom sediments, in the first stage, they were initially dried and ground and dried to a constant weight at 550°C according to PN‐88/B04481 (Ostrowska, Gawliński, and Szczubiałka 1991). After burning, the content of organic matter in the bottom sediments was determined using the loss on ignition (LOI) method, in which weight loss in bottom sediment samples is measured and calculated [%]. The criteria for its interpretation were adopted after Verdonschot (2001): > 10%—very high content of organic matter; 4%–10%—high content of organic matter; 1%–4% average content of organic matter; < 1%—low content of organic matter.

### 2.6 Statistical Analysis

We carried out a multivariate model of Canonical Correspondence Analysis (CCA) to explore the data on aquatic invertebrate occurrence combined with the environmental variables to visualize their distribution pattern in artificial forest lakes. Benthos data were log (x + 1) transformed and unimodality tested using a detrended correspondence analysis (DCA), which confirmed that the data were unimodal and the gradient length > 3.0 (Ter and Verdonschot 1995). We used manual forward selection and Monte Carlo permutation test, carried out for 499 replicates to analyze significant differences between taxa, environmental factors, and lakes (Legendre and Legendre 2012), including unrestricted permutation to select the environmental factors that had a strong influence on the benthos composition. The environmental variables with a high inflation factor were excluded from the final CCA analysis in forward selection. The ordination diagram represents the pattern of community distribution and the main features of the distribution of the taxa along the environmental variables (CANODraw, CANOCO 4.5 software). Analyses were performed using CANOCO for Windows 4.5 (Ter and Šmilauer 2002).

We used Spearman rank correlation coefficients (r_s_) to analyze the relationships between the environmental factors and benthic invertebrate occurrence (STATISTICA version 13.1; Dell version). The diversity indices (Shannon‐Wiener, Simpson, and Pielou Evenness Index) were calculated using the MVSP (3.13.p Kovach Computing Services).

## 3 Results

### 3.1 Assessment of Ecological Status and Macrophyte Diversity in Artificial Forest Lakes

**FIGURE 2:**
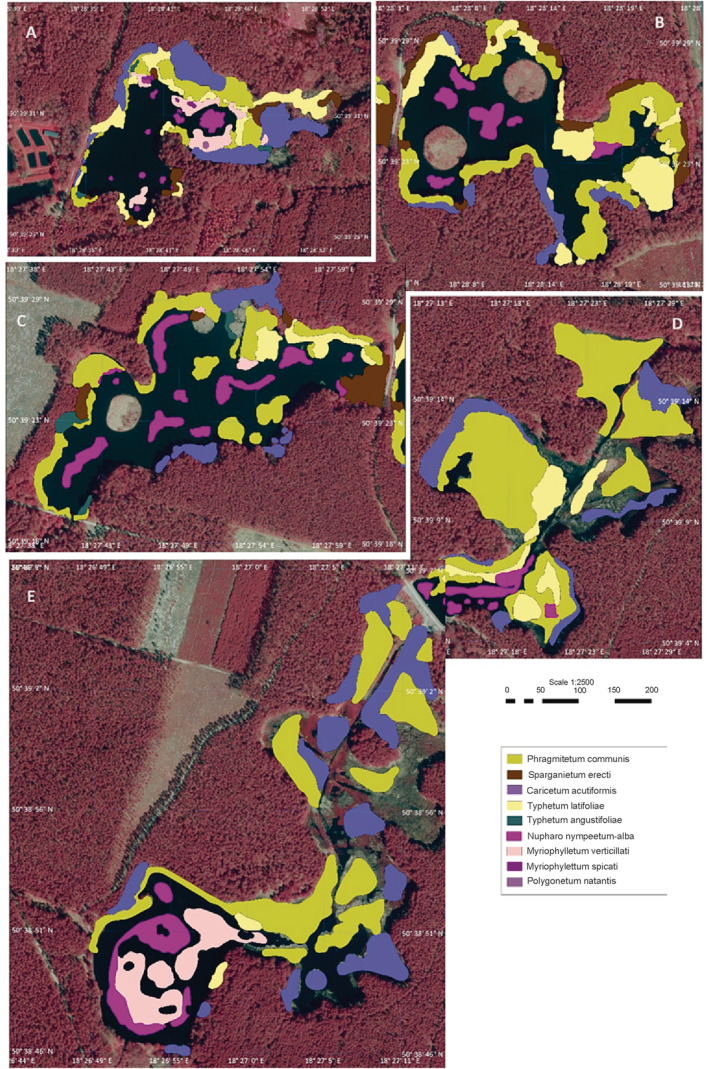
Model of plant associations in the studied artificial forest lakes (A) lake 1, (B) lake 2; (C) lake 3; (D) lake 4; (E) lake 5.

The aquatic vegetation of the studied complex of forest lakes found at the sampling points is presented in Table 2. In total, 24 species of plants were found. The largest number of species was recorded in lake 2 and the least in lake body 4. Among them, the species covered by partial protection in Poland, for example, *Nuphar lutea* and *Nymphaea alba* , were found, as well as the species of carnivorous plant *Utricularia vulgaris* . Aquatic plants in the studied complex are diverse. Plants inhabit the open water, and in the riparian zone, they form vegetation patches characteristic of low or transitional types of peat bogs. The species overgrow the parts of lakes that have undergone the process of overlanding as a result of being filled with organic and mineral material. The distribution and ranges of all identified phytocoenoses are presented on the maps (Figures 1 and 2).

**TABLE 2:** Aquatic macrophytes of the studied complex of forest lakes. 1–5—the number of forest lake.

Species	1	2	3	4	5
*Nuphar lutea* (L.) Sibth. & Sm.		x	x	x	x
*Nymphaea alba* (L.)			x		x
*Sparganium erectum* (L.)		x	x		
*Lycopus europaeus* (L.)	x	x		x	
*Iris pseudacorus* (L.)		x			
*Glyceria aquatica* (L.) Wahlenb.	x		x		x
*Mentha aquatica* (L.)		x	x		x
*Schoenoplectus lacustris* (L.) Palla					
*Hottonia palustris* (L.)			x		
*Typha latifolia* (L.)		x			
*Typha angustifolia* (L.)		x			
*Utricularia vulgaris* (L.)			x		
*Eleocharis palustris* (L. Roem. & Schult.)		x		x	
*Eleocharis acicularis* (L.) Roem. & Schult.				x	
*Solanum dulcamara* (L.)	x	x			
*Lemna minor* (L.)			x		
*Scirpus sylvaticus* (L.)	x				
*Acorus calamus* (L.)		x			
*Lysimachia nummularia* (L.)					x
*Phragmites australis* (Cav.)Trin. ex Steud		x	x	x	x
Carex sp.	x	x		x	x
*Eriophorum angustifolium* Honck.				x	
*Myriophyllum spicatum* (L.)					x
*Myriophyllum verticilatum* (L.)		x			x
*Alisma plantago aquatica* (L.)			x		
Number of taxa	5	13	10	7	9

In lake 1, nine plant associations were observed (Table 3). The phytolittoral occupied 68% of the area, which is the highest result among the studied complex. Plants with floating leaves had the smallest percentage in the phytolittoral—19%, submerged plant communities also occupied a small area—24%, and carex rushes—35% dominated over other rushes (22%). *Carex acutiformis* formed single‐species rushes covering almost most of the shores and overland area; it locally co‐occurred with *Carex pseudocyperus* .

**TABLE 3:** Plant associations in the phytolittoral zone of artificial forest lakes.

Plant assemblage	1	2	3	4	5	In total
Reed communities—*Phragmitetea* class of associations						
*Phragmitetum australis*	20.5%	41.1%	42.0%	64.7%	42.4%	45.1%
Carex L.	33.8%	9.6%	13.4%	17.1%	31.1%	21.4%
*Typhetum angustifoliae*	1.2%	0.0%	3.7%	0.0%	0.0%	0.7%
*Typhetum latifoliae*	21.0%	29.6%	9.0%	12.7%	1.5%	12.7%
*Sparganietum erecti*	6.3%	10.2%	11.3%	0.0%	0.0%	4.3%
Rushes in total	82.9	90.5	79.4	94.5	75.0	84.5
Submerged communities—*Potametea* class of associations						
*Nupharo‐Nymphaeetum albae*	4.9%	9.5%	19.4%	5.6%	8.7%	9.3%
*Myriophylletum verticillati*	11.2%	0.0%	1.3%	0.0%	13.9%	5.8%
*Myriophylletum spicati*	0.7%	0.0%	0.0%	0.0%	0.0%	0.1%
*Polygonetum natantis*	0.3%	0.0%	0.0%	0.0%	2.3%	0.7%
Submerged plant in total	17.1	9.5	20.7	5.6	24.9	15.9

Lake phytolittoral occupied 51% of the lake's surface. Dominating 90.5% part of the phytolittoral constitutes the rushes, consisting of mainly the 3 species *Sparganium erectum* , *Typha latifolia* , and *Phragmites australis* . Submerged species of plants co‐occurred with the species with floating leaves—9.5%. The only class of association observed in this category was *Nupharo‐Nymphaeetum albae*.

The phytolittoral in lake 3 was the smallest in percentage of the total lakes surveyed, occupying only 45% of the lake area (Table 3). In this lake, large and single‐species clusters were formed by common reed *Phragmites australis* , which covered the phytolittoral in 42%. Plants with floating leaves occupied 19.4%, and carex rush only 13.4%. An interesting phenomenon is the formation of a geometrically homogeneous strip of 3.5 m wide of this species, which separated the reservoir from the forest trees in the riparian zone.

The phytolittoral of lake 4, occupied 64% of the present lake area (Figure 2, Table 3). Reed plant communities constituted 94.4% of the phytolittoral, of which 65.5% were carex rushes. The floating plant communities in this lake were exclusively represented by Nupharo‐Nymphaeetum albae, which constitute 5.6% of the phytolittoral.

Lake 5, compared to other lakes, is distinguished by a large percentage of submerged plant communities (13.9%) of the phytolittoral, which is dominated by myriophyllum, 11.1% constitute plant communities with floating leaves, 44% rushes, and 31% carex rushes (Table 3). Studies revealed the negative relationship of rushes with the organic matter content in the bottom sediments of the lakes (*r*_s_ = −0.741, *p* < 0.05) and positive with submerged associations of plants (*r*_s_ = 0.742, *p* < 0.05).

The total area of phytolittoral, lake surface, and number of communities forming a phytolittoral was diverse in the studied complex (Table 4). The occupation index values were the highest in lakes 1 and 4, whereas the values of biocenotic diversity and maximal biocenotic indices were the highest in lake 1 (Table 4). The assessment of the ecological status of lakes using macrophyte index showed that water achieved good ecological status (II Quality Class) (Tables 4 and 5).

**TABLE 4:** The ecological indices measured for the plant communities in lakes.

Indices		1	2	3	4	5
Ecological state macrophyte index	ESMI	0.648	0.526	0.416	0.585	0.481
Biocenotic diversity index	H	1.692	1.407	1.593	1.007	1.366
Number of communities forming a phytolittoral	S	9	5	7	4	6
Maximal biocenotic diversity	H_max_	2.197	1.609	1.946	1.386	1.792
Occupantion index	Z	0.684	0.512	0.428	0.639	0.514
Total area of the phytolittoral	N	2.68	4.062	3.501	5.742	7.023
Lake surface	P	3.92	7.93	8.17	8.99	13.66
Phytolittoral/surfaces	N/P	68%	51%	43%	64%	51%
Redundancy indicator	R	0.230	0.126	0.182	0.274	0.237

**TABLE 5:** The quality class of the ecological status/potential of lakes. According to ESMI Macrophyte Ecological Status Index for non‐saline lakes (Ciecierska et al. 2006).

Ecological status/potential	Value of ESMI
Very good	≥ 0.680
Good	≥ 0.410
Moderate	≥ 0.205
Poor	≥ 0.070
Bad	≥ 0.070

In the ponds studied, the proportion of floating plant communities varied from 5% in lake 4 to 19.4% in lake 3, with an average of 10% of the phytolittoral (Figure 2). Submerged plant communities were only found in Lakes 1, 3, and 5, and their total contribution to the phytolittoral was small, not exceeding 6%. In the case of all the ponds, the Riparian plant communities accounted for more than 75% of the phytolittoral area. The average for the pond complex was 84%.

### 3.2 Water Chemistry in Artificial Forest Lakes

The analysis of the physicochemical properties of water showed low values of salinity indicators such as conductivity, TDS, the concentration of chlorides and sulfates in all lakes in this study. The pH of the water ranged from 6.2 to 7.6; therefore, it was slightly acidic or within the limits of neutral pH values. The content of dissolved oxygen was quite high and showed slight differences in particular months of sampling (Table 6). The content of nitrogen and phosphorus was low in all of the lakes. The relatively high content of iron was surprising in some lakes. Their water was soft.

**TABLE 6:** Water chemistry and the content of organic matter artificial lakes.

		1	2	3	4	5
Temperature	°C	18–25	17.9–25.8	18.1–25.8	19.1–25	20.8–25.8
Odczyn	pH	6.2–7.6	6.5–7.5	6.8–7.4	6.5–7.3	6.6–7.4
EC	μS/cm	180–230	180–290	192–220	180–270	180–240
TDS	mg/dm^3^	80–110	80–130	90–100	80–120	80–110
DOX	mg/dm^3^	7.62–10.49	6.96–10.69	3.34–8.40	6.5–9.1	7.28–40.48
Hardness	mgCaCO_3_/dm^3^	110–140	110–155	114–148	120–150	110–152
Alkalinity	mgCaCO_3_/dm^3^	55–75	60–80	60–100	50–120	50–125
Chlorides	mg/dm^3^	22–30	20–38	24–50	18–38	22–32
Calcium	mg/dm^3^	32–62	32–85	30–70	32–78	30–68
N‐ NH_4_	mg/dm^3^	0.51–0.91	0.44–0.81	0.46–0.55	0.39–0.89	0.38–0.67
NH_3_	mg/dm^3^	0.62–1.24	0.53–0.98	0.56–0.66	0.47–1.03	0.46–0.85
N‐NO_3_	mg/dm^3^	0–0.7	0–1.0	0	0.1–0.9	0.3–0.7
NO_3_	mg/dm^3^	0–3.1	0–4.43	0	1.9–17.6	1.32–3.1
N‐NO_2_	mg/dm^3^	0	0–0.005	0–0.005	0–0.002	0
NO_2_	mg/dm^3^	0	0–0.02	0–0.02	0–0.006	0
PO_4_	mg/dm^3^	0.09–0.37	0–2.5	0.06–1.22	0–1.33	0.15–0.28
SO_4_	mg/dm^3^	2.10–2.45	1.8–2.48	2.10–2.48	28–31	27–29
Fe	mg/dm^3^	1.20–24	0.86–29	0.93–29	0.31–0.65	0.07–0.3
Average OMC	%	21.84	2.53	25.3	0.65	14.04

Abbreviations: DOX, dissolved oxygen; EC, conductivity; OMC, organic matter content; TDS, total dissolved solids.

### 3.3 The Content of Organic Matter in the Bottom Sediments of Forest Lakes

The content of organic matter in the bottom sediments of the artificial lake complex was different. Its highest content, which, according to Verdonschot (2001), can be classified as very high, was found in sediment samples collected in lakes 1, 3, and 5. In the other two lakes, it was low and amounted to 2.53% (average content of organic matter) and 0.65% (low content of organic matter) in lakes 2 and 4, respectively (Table 6).

### 3.4 Benthos Invertebrate Diversity Assessment in Artificial Forest Lakes

The benthic fauna in the complex of studied forest lakes is diverse; however, both the number of taxa and the density of invertebrates differ in individual seasons and between lakes. The largest number of individuals were collected in lake 4; the highest average density of benthos was also recorded in this lake—3235 ind./m^2^. Similarly to the number of collected specimens, the smallest density was found in lake 2 (Table 7).

**TABLE 7:** The mean density [ind./m^2^] of benthic invertebrates in forest lakes.

Takson	1	2	3	4	5
Oligochaeta	67	17	11	555	335
Crustacea *Asellus aquaticus*	0	3	0	5	2
Nematoda	0	0	0	8	1
Hirudinea					
*Helobdella stagnalis*	17	1	0	45	5
*Hemiclepsis marginata*	9	0	0	8	1
*Erpobdella nigricolis*	109	0	0	5	0
*Erpobdella octoculata*	0	0	0	1	1
*Glossiphonia heteroclita*	0	0	1	5	0
*Glossiphonia complanata*	0	0	0	1	0
*Haemopis sanguisuga*	0	0	0	1	0
Insecta					
Odonata					
Lestidae	11	11	13	15	11
Libellulidae	4	0	1	8	3
Cordulidae	0	1	0	21	0
Coenagrionidae	0	8	1	40	30
Platycnemidae	0	5	0	36	5
Ephemeroptera					
Caenidae	17	7	7	324	84
Betidae	0	16	3	184	32
Siphlonuridae	0	0	0	0	1
Trichoptera					
Leptoceridae	9	15	1	128	12
Ecknomidae	4	21	0	1	14
Hydropsychidae	1	0	0	0	0
Hydroptilidae	0	1	3	12	1
Polycentropodidae	0	0	0	15	9
Phryganeidae	0	0	0	9	5
Diptera					
Chironomidae	967	603	200	1664	178
Tabanidae	8	1	0	4	1
Ceratopogonidae	3	4	3	9	1
Limonidae	1	1	0	1	0
Chaoboridae	0	1	0	0	0
Dixidae	0	0	1	0	0
Coleoptera					
Helodidae	1	0	0	0	0
Dytiscidae	0	0	20	0	4
Haliplidae	0	0	1	0	0
Hydrophilidae	0	0	1	9	0
Heteroptera					
*Mesovelia furcata*	0	1	0	0	0
*Ranatra linearis*	0	1	0	0	0
*Ilyocoris cimicoides*	0	0	3	5	0
*Nepa cinerea*	0	0	0	0	7
*Plea minutissima*	0	0	1	0	0
Corixidae	0	0	3	8	0
Megaloptera					
*Sialis lutaria*	0	0	1	4	0
Neuroptera					
Planipenia	0	0	0	1	0
Gastropoda					
*Stagnicola palustris*	0	1	0	0	0
*Anisus vortex*	4	4	5	0	0
*Anisus spiroribis*	0	0	1	0	0
*Gyraulus albus*	0	11	3	24	40
*Gyraulus crista*	0	0	1	3	0
*Planorbarius corneus*	0	0	0	5	34
*Hippeutis complanatus*	0	8	13	29	1
*Segmentina nitida*	0	0	1	1	0
*Radix balthica*	—	4	0	1	5
*Acroloxus lacustris*	0	0	0	9	1
*Ferrissia fragilis*	3	1	0	11	3
Bivalvia					
*Sphaerium corneum*	3	0	0	1	0
*Pisidium casertanum*	0	0	4	3	0
*Musculium lacustre*	0	0	0	3	0
The average density of benthos in a pond [ind/m^2^]	1237	755	311	3235	859
Number of taxa	17	28	27	42	29
Simpson diversity index	0.38	0.34	0.56	0.68	0.77
Pielou Evenness index	0.40	0.36	0.58	0.70	0.80
Shannon‐Wiener diversity index	0.95	1.02	1.59	1.77	2.03

In lake 1, a total of 17 taxa were found, among which the highest density had larvae of the Chironomidae family. Apart from Chironomidae also, a high density of leeches, *Erpobdella nigricolis*, and oligochaetes were found in this lake; the other taxa achieved relatively low densities. In lake 2, a total of 28 taxa of benthic invertebrates were indicated, which, with the exception of Chironomidae larvae, were characterized by low density. In the fauna of freshwater snails, the occurrence of 6 species was found, including one also *Ferrissia fragilis* (Table 7). In lake 3, a total of 26 taxa of invertebrates were found, among which Diptera larvae were the most numerous. In the fauna of freshwater snails, the presence of 8 species was found; however, they achieved low densities, similar to other taxa found (Table 7). In lake 4, a total of 42 taxa with different densities were found. There were numerous Oligochaeta and larvae of insects (Chironomidae). In the fauna of leeches, the presence of 7 species was found, of which *Helobdella stagnalis* has the highest density (Table 7). In the mollusk fauna, there were 8 species of snails and 3 species of mussels. Regarding the number of taxa and their density, the fauna of benthic invertebrates in this lake was rich and diverse. In the context of biodiversity, the structure of invertebrate fauna in lake 5 was diverse and can be described as rich. The analysis of the study results showed the presence of 35 taxa of invertebrates. Quite high density was reached by oligochaeta (Oligochaeta), larvae of mayflies from the family Caenidae, Chironomidae, dragonflies (Odonata), and snails (Gastropoda). The values of diversity indices indicate good environmental conditions of lakes (Table 7).

**FIGURE 3:**
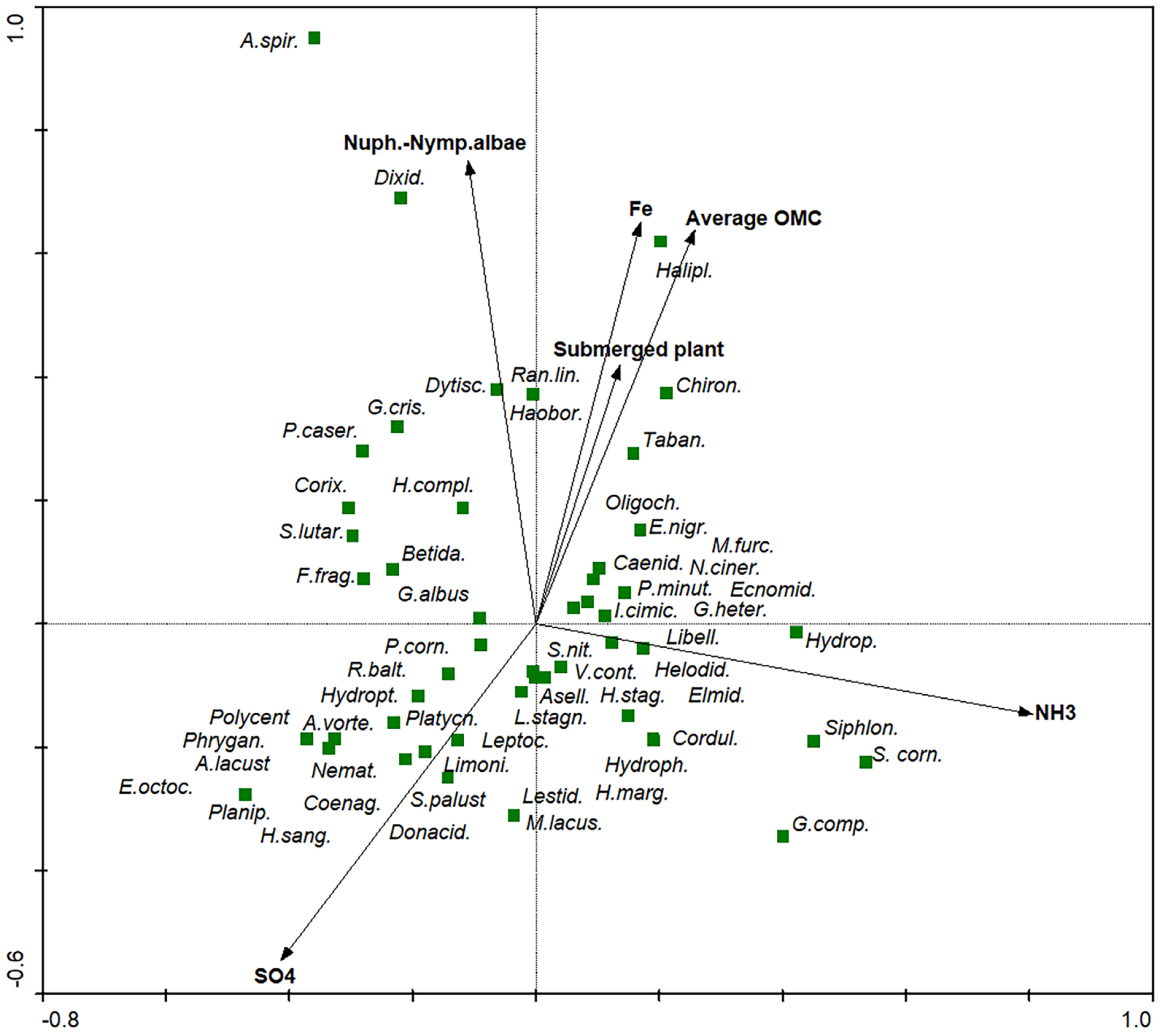
The diagram of ordination space in the CCA analysis. Average OMC: average organic matter content in the bottom sediments of lakes; Nuph.‐Nymp.albae, *Nupharo‐Nymphaeetum albae* plant communities.

The CCA ordination diagram showed the location of taxa in the ordination space of axes I and II and the directions of the intensity of the changes in the size of environmental variables. The results of the multivariate CCA analysis showed that the iron content in water and the organic matter content in the bottom sediments are significantly related to the distribution of selected groups of benthic organisms in forest lakes (Figure 3). In the lower left part of the ordination diagram, taxa characteristic for low values of iron in water and organic matter in bottom sediments are placed. The occurrence of these taxa was also related to the presence of submerged plants at sampling sites. The occurrence of other benthos taxa was associated with higher NH3 and SO4 content in the water (Figure 3). Statistical significance Monte Carlo test: first axis: *F* = 5.247, *p* = 0.002; all canonical axes: *F* = 3.281, *p* = 0.002. Axis 1 explains 26.2% of the overall variability of benthic invertebrates, (Axis 2 14.1%—Cumulative percentage variance of species‐environment relationships).

## 4 Discussion

### 4.1 Macrophytes Assessment of Small Artificial Forest Lakes

Aquatic macrophytes can serve as useful indicators of water pollution along the littoral of stagnant waters. Research on macrophytes in lakes has resulted in the development of a wide range of metrics indicating their ecological conditions (Stelzer, Schneider, and Melzer 2005; Ciecierska and Dynowska 2013). The macrophyte assessment of the studied complex of forest lakes showed variability of environmental conditions between the five investigated objects. The structure of their phytolittoral is clearly differentiated (Figure 2) even though they constitute a hydrographic and hydrological wholeness, and the geological and natural conditions are homogeneous. The highest value of the ESMI index was recorded in lake 1–0.648 and the lowest in lake 3–0.416. The ESMI index ranges from 0 to 1 (Kolada, Ciecierska, and Bociąg 2020), where 0 means a degraded state and 1 means the state closest to natural. The values calculated for the lakes in the studied area are ranked in the middle of the scale, which can be interpreted as a transformed but good condition. The limit values of the ecological status classes of the ESMI index for inland hard‐water lakes were established based on actual ESMI values in the database used to develop the method (Ciecierska et al. 2006; Portielje et al. 2014). Based on the obtained results, the ecological status of lakes was classified, which allowed us to indicate that all forest lakes can be classified as having good ecological status.

The manifold role of freshwater macrophytes in stagnant water ecology is closely linked to their distribution and biomass, which constitute a synergy of various environmental factors (Nurminen 2003). In the context of the taxonomic composition of macrophytes, in the phytolittoral of lakes, a significant difference between the taxonomic composition of the communities was noticed. In lakes 2 and 4, over 90% of the phytolittoral belongs to reed communities, with a dominant percentage of *Phragmitetum australis*, Carex, and *Typhetum latifolia*. The smallest percentage of reed communities was recorded in lakes 5 and 3—below 80%. Submerged plant communities were the most numerous in lake 5 and the least numerous in lake 4. This difference is visible and important because these lakes are adjacent to each other (Figure 2). This probably results from the dominance of the reed community in the lake, which constitutes 94.5% of the phytolittoral area. The control studies on the impact of littoral slope, location, and wind exposure in shallow shore areas on the ESMI values change (Kolada, Kutyła, and Bielczyńska 2024), indicated that lakes with a higher littoral slope and wind exposure were characterized by a higher ecological status than transects with a hidden slope that was sheltered from the wind. The investigated forest lakes are shallow and have a small surface area, located in the forest interior. Such conditions may impact the ESMI values, indicating poorer ecological status.

The rush vegetation, along with submerged and floating leaved plants, which are a frequent component of vegetation cover of most stagnant freshwater bodies, usually creates the first transitional zone of land‐water vegetation that overgrows the shallow parts of littoral (Podbielkowski and Tomaszewicz 1996). In this study, the reed zone of lakes is very well developed and is present practically along the entire shoreline. Our results showed that the dominant reed community is the reed of *Phragmitetum australis*, which constitutes as much as 45% of the phytolittoral area of the entire complex of artificial lakes. Furthermore, the community of the *Caricetum acutiformis* and the *Sparganietum erecti* occupy an area of 21.4% and 12.7% of the phytolittoral, respectively. These communities occur in all lakes studied. Compact and well‐developed rush vegetation constitutes a protective barrier against the flow of pollutants from the land (Stachowicz and Nagengast 2010; Łaskawiec 2015). Large patches of reed patches limit the expansion of other communities and reduce diversity. Reed stands to provide habitats for nesting, feeding, or roosting of vulnerable bird species (Čížková et al. 2023). Aquatic submerged vegetation constitutes 15.9% of the total phytolittoral of the studied lakes. The largest and best‐developed community is *Nupharo‐Nymphaeetum albae*, constituting 9.3% of the phytolittoral area. It was the only one inventoried in all artificial lakes. As is clear from the study of Joniak, Kuczyńska‐Kippen, and Nagengast (2007), small lakes create specific ecosystems in which seasonal changes in the temperature and chemical conditions reflect the dynamic balance between organisms and the environment in relation to numerous influential external factors.

Globally observed trends of biodiversity loss in small lakes are mainly due to several complex factors affecting their ecological structure and function. Studies by Janse et al. (2015) and Bolpagni et al. (2019) show that small water reservoirs, are decreasing their capacity for nutrient retention and ecological stabilization under the influence of increased pollution and eutrophication. Urbanization and hydrological changes lead to the degradation of aquatic ecosystems (Vörösmarty et al. 2010). The lakes analyzed, located in forested areas, are subject to limited anthropopressure despite their artificial origin. As a result, they play a key role in maintaining biodiversity and ecological stability in a region where natural reservoirs are not available (Figure 1).

The main threats to small artificial lake ecosystems also include climate change (Moss et al. 2009; Čížková et al. 2013; Taniwaki et al. 2017) and invasion by foreign species (Riley et al. 2018). The main threat to lakes studied is the reduction in surface area and volume of the reservoirs, which results in the loss of their natural functions and the loss of habitat for species adapted to constant hydrological conditions. This results in a reduction of biodiversity and changes in ecosystem structure (Jurik et al. 2015; Riley et al. 2018) or extinction of species sensitive to hydrological changes (Moss et al. 2009; Eissa and Zaki 2011;  Miranda et al. 2020).

Climate change has a significant impact on the biodiversity of small reservoirs, with consequences closely linked to rising temperatures, hydrological changes, and the increasing frequency of extreme weather events. Studies show that climate warming and droughts lead to reduced flows in small watersheds, which reduce water retention times, decrease oxygen levels, and increase pollutant concentrations. The result is a deterioration of living conditions for many species, which can result in their extinction or migration. In conclusion, predicted climate change may accelerate the loss of biodiversity of small reservoirs. In order to counteract these effects, sustainable management of these resources is needed, taking into account, among other things, pollution reduction and protection of local species. The conclusions also highlight the urgent need to introduce the creation and protection of small lakes, which play a key role in maintaining biodiversity and ecological stability.

### 4.2 Biological Diversity of Studied Complex of Forest Lakes in the Context of Changing Climatic Conditions

Global climate change has been identified as a main driver affecting aquatic ecosystems (e.g., Vescovi et al. 2009; Van Beek et al. 2011), including a rise in water temperature and hydrological changes. Our results from the artificial lakes studied relate to global trends in biodiversity loss or small retention water bodies. They can be scaled up to other aquatic environments that originated as human‐induced but are old enough to be treated as a constant element of the landscape. Their role in the small retention is important, but it can depend on land‐use changes, which indirect effects include increased erosion after deforestation (Wissmar et al., 2004), as well as with eutrophication because biogenic concentrations usually correlate with the intensity of land use (Crosbie and Chow‐Fraser, 1999). A study by Janse et al. (2015) shows that biodiversity intactness in freshwater ecosystems is negatively related to two dominant categories of anthropogenic stressors. The first is land use and eutrophication in the catchment (affecting “water quality”), and the second is hydrological disturbance by dams and climate change. Whereas many studies have investigated climate‐induced effects on the phenology and abundance of single species, less is known about climate‐driven shifts in the diversity and composition of entire communities (Burgmer, Hillebrand, and Pfenninger 2007). Climate change may negatively impact biodiversity, leading to population declines, loss of genetic diversity, changes in the geographic distribution of species, and, consequently, species extinction. As was shown by Weinert et al. (2022), benthic bioturbation is one of the most relevant and significant processes for benthic‐pelagic coupling in marine sediments. Also, in freshwater ecosystems, benthic organisms can be an indicator of changing conditions of environments. Therefore, climate‐induced shifts of species and, consequently, the alterations in the composition of benthic assemblages might eventually affect ecosystem processes, for example, by the altered community structures. The long‐term studied forest lakes' existence enabled us to undertake research on the importance of human‐made lakes located in forests in the context of small retention and biodiversity in the context of climatic changes.

The location of the lakes relative to the hydrological network can influence the water pH; for example, if located in the watershed divide zone, they may have a lower pH values, which means that benthic invertebrate communities also have a different structure (Walker, Fernando, and Paterson 1985; Rodrigues and Scharf 2001; Spyra, Cieplok, and Kaszyca‐Taszakowska 2023). However, in this study, we found that the pH of the water ranged from 6.2 to 7.6; therefore, it was slightly acidic or within the limits of neutral pH values. Activities undertaken in the forest catchment affect the chemistry of lake waters. The changes in the water chemistry showed that the forestry treatments, especially the soil scarification, increased both the organic and the inorganic load from the catchment to the lake (Walker, Fernando, and Paterson 1985). The physicochemical parameters of the water in human‐made water bodies with similar morphometry, origin, and surroundings are comparable to our results. For example, in the forest Lake Piaseczno, the water pH did not exceed 8.2, the conductivity ranged between 61.06–98.52 μS/cm, and the content of nutrient concentration was very low (Radwan and Kowalczyk 1979). We showed low values of salinity indicators such as conductivity, TDS, the concentration of chlorides and sulfates. The content of dissolved oxygen was quite high and showed slight differences in particular months of sampling, whereas the content of nitrogen and phosphorus was low in all of the lakes. Surprising was the relatively high content of iron in some reservoirs.

Organic matter has an impact on the structure of aquatic invertebrate communities in different types of environments. It is high or very high in environments with rich aquatic vegetation or a large amount of organic debris (e.g., allochronic plant matter in the form of leaves fallen from trees into water) and poor in aquatic environments with a small amount of debris and a sandy bottom. The content of the organic matter in the bottom sediments of the forest reservoirs of this study reached 25% in some lakes, indicating an organic matter‐rich environment. These are the values known from those obtained in other studies of non‐artificial lakes located in forests (Trojanowski and Antonowicz 2005; Rafałowska and Sobczyńska‐Wójcik 2014). The fluctuation in values is most likely related to the values of submerged communities in the *Potametea* class of associations, which were the lowest in lakes with the lowest content of the organic matter in the bottom sediments of forest lakes studied. Plants within lakes can affect the distribution and diversity of aquatic invertebrate communities (de Szalay and Resh 1996; Meyer, Peterson, and Whiles 2011); however in our study, we noted the occurrence of different numbers of plant species in particular forest lakes, which can be a probable factor for various density of mollusks.

The studies on the structure of benthos assemblages are carried out in various types of reservoirs, while natural ones surrounded and overgrown by dense vegetation are often inhabited by numerous aquatic organisms because their surroundings most often have a positive effect on communities. Benthic density can be a useful indicator of ecosystem quality in relation to the number of benthic taxa. Research in a similar type of water bodies was carried out in the Uściwierz forest lake, where the occurrence of 26 taxa was found, of which the most numerous were Chironomidae, represented by 18 species. The average benthic density was 3000 individuals/m^2^ (Radwan et al. 2003). Studies by Michałkiewicz (1991) found 16 taxa in Lake Lednickie, the most numerous of which were freshwater snails, and the average density of benthos was 2148 individuals/m^2^, similar to those obtained by Radwan et al. (2003). Research conducted in Russia in a large, partially forested Arakhley reservoir revealed the presence of 27 taxa (Matafonov 2021), and the most numerous were also chironomid larvae (41%), while in the Ristonlampi mid‐forest reservoir, located in Finland, the presence of only 6 taxa was found, such as Ephemeroptera, Odonata, Trichoptera, Coleoptera, Neuroptera, and Chironomidae, which dominated the benthic fauna, with the percentage reached 80%. The average density of benthos in this lake, despite the small number of taxa, was almost 14,000 individuals/m^2^ (Rask et al. 1998). In lake 1 of this study, a total of 17 taxa were found, among which the highest density of Chironomidae was found. The lower diversity of fauna in comparison with the other reservoirs is most likely due to the larger depth of lake 1 at the sampling site; moreover, in this lake, fish stocking is carried out, which may affect the number of taxa. In this study, we found the average density of benthos the highest in lake 4 (3235 ind/m^2^), and the number of benthos taxa varied from 17 to 42. Some invertebrates can endure pronounced and unpredictable changes in the environment; hence, they likely possess a potential tolerance to most natural variations in the habitat and environmental conditions (Batzer 2013), however, others are fragile to environmental changes. A study by Corline et al. (2022) demonstrated the formation of a unique invertebrate community populated by lentic invertebrates, which increased diversity. According to our results, the diversity of benthic fauna can indicate the local diversity of forest lakes, especially in water fluctuation.

While aquatic organisms can adapt to changing ecosystems, predicting how individual species will respond to climate change is quite a complex process. Studies by Weiskopf et al. (2020) found that species respond to climate change by changes in morphology and behavior, phenology, and geographic range shifts. Responses by species and populations, along with direct effects of climate change on ecosystems (including more extreme events), are resulting in widespread alterations in productivity, species interactions, vulnerability to biological invasions, and other emergent properties (Weiskopf et al. 2020).

Data from various reports, including the Global Assessment Report on Biodiversity and Ecosystem Services (IPBES 2019), indicate that currently, the rate and scale of species extinction in the world is very rapid. Therefore, biodiversity is in a critical situation globally, as a consequence of ongoing climate change caused by human activity. European Environmental Agency (EEA) in the report “Europe Environment 2020 – status and predictions” (SOER 2020) indicates an unfavorable conservation status for 60% of species and 77% of habitats protected under the Environment Directive. Biodiversity loss is not limited only to rare or threatened species. According to IUCN (Foden and Young 2016), the effects of climate change threaten many species around the world, and sensitivity analyses conducted by scientists suggest that even more species may not be able to adapt to new climatic conditions. The most sensitive to climate change are plant and animal species associated with water ecosystems and mountain habitats, as well as with natural forests. Various human‐made threats are changing rapidly over time, emphasizing the need to monitor future trends of all threats, including climate change (Woo‐Durand et al. 2020). For this reason, lakes created in forest complexes are important for the biological diversity of these areas on a global scale. Our knowledge is still incomplete on how the observed changes may evolve under different climate models, however, since lakes increase water retention in forests with the ongoing climate warming, they will most likely threat threaten to disappear due to decreasing water levels, drying out, and overgrowing as a result of increasing temperature.

## 5 Conclusions

The investigated small forest lakes are artificial water environments where periodic activities are carried out to maintain the infrastructure regulating water flow. Despite these activities, lake condition, as assessed by the ESMI index or the biocenotic diversity index, is good. The noteworthy is the high percentage of reed communities, which are less favorable in the context of biocenotic diversity. Climate change, expressed by an increase in the frequency of dry years, creates a situation of changes in the level of filling lakes with water, which, taking into account their small depth, results in dynamically changing conditions for the development of phytolittoral. Comparing our results from 2021 to aerial photos from 2019 (Figure 1), significant differences in the range of plant communities can be seen (Figure 2). Investigating the range of occurrence of plant communities in subsequent years would allow determining the rate and direction of these changes. Along with the phytolittoral changes, benthic invertebrate communities change, their density fluctuates, and the number of taxa also fluctuates. It should be assumed that with ongoing climate change, these phenomena will intensify, which will lead to changes in entire ecosystems at both the plant and animal levels. Finally, we suggest avenues of research required to address current gaps about the climate‐induced effects on freshwater lakes, including the need for more long‐term data analyses, focusing on the distributional approaches, including also various non‐climatic stressors as an emerging problem with global implications.

## Author Contributions

**Rafał Starzak:** conceptualization (equal), data curation (equal), formal analysis (equal), methodology (equal), software (equal), validation (equal), visualization (equal), writing – original draft (equal), writing – review and editing (equal). **Anna Cieplok:** conceptualization (equal), data curation (equal), formal analysis (equal), methodology (equal), software (equal), validation (equal), visualization (equal), writing – original draft (equal), writing – review and editing (equal). **Robert Czerniawski:** formal analysis (equal), writing – original draft (equal), writing – review and editing (equal). **Aneta Spyra:** conceptualization (equal), data curation (equal), visualization (equal), writing – original draft (equal), writing – review and editing (equal).

## Conflicts of Interest

The authors declare no conflicts of interest.

## Supporting information


Appendix S1.

## Data Availability

The data generated during this study are available Supporting Information.
