# Correction to “The Role of Artificial Lakes Located in Forests in the Context of Small Retention, Biodiversity and Climatic Changes—Evidence From Southern Poland”

**DOI:** 10.1002/ece3.70980

**Published:** 2025-03-16

Starzak, R., Cieplok, A., Czerniawski, R. and Spyra, A. (2025), The Role of Artificial Lakes Located in Forests in the Context of Small Retention, Biodiversity and Climatic Changes—Evidence From Southern Poland. *Ecol Evol*, 15: e70775. https://doi.org/10.1002/ece3.70775

The funding statement in the original article was published as:

Funding: This research was funded by the University of Agriculture in Krakow and the University of Silesia in Katowice. The research was partially also financed under the grant “Development of a strategy for managing migratory salmonids in a restored river with an indication of ways to protect them, the example of the Drawa River” by the European Union from the European Maritime and Fisheries Funding included in the Operational Program Fisheries and Sea 2014–2020 (Agreement no. 0007‐6520.13‐OR1600001/22/23).

The correct statement is:

Funding: This research was funded by the University of Agriculture in Krakow and the University of Silesia in Katowice. The research was partially financed by the Minister of Science under the “Regional Excellence Initiative” Program. Agreement No. RID/SP/0045/2024/01.

We apologize for this error.

