# Supplementary material for: The Role of Artificial Lakes Located in Forests in the Context of Small Retention, Biodiversity and Climatic Changes—Evidence From Southern Poland
Source: Ecol Evol. 2025 Jan 21;15(1):e70775. doi: 10.1002/ece3.70775 (PMC11747350; doi:10.1002/ece3.70775)
Supplement: Supplementary file 1 — Appendix S1. [file ECE3-15-e70775-s001.docx]

**APPENDIX**

**SUPPLEMENTARY MATERIAL**

**The role of artificial lakes located in forests in the context of small retention, biodiversity, and climatic changes – evidence from southern Poland**

**Rafał Starzak^1^, Anna Cieplok^2^*, Robert Czerniawski^3^, Aneta Spyra^2^**

^1^ Department of Ecological Engineering and Forest Hydrology, University of Agriculture in Krakow, Al. 29 Listopada 46, 31-425 Kraków, Poland; ^2^ Institute of Biology, Biotechnology and Environmental Protection, Faculty of Natural Sciences, University of Silesia, 9 Bankowa Str, 40-007 Katowice, Poland; ^3^ Department of Hydrobiology, Institute of Biology, University of Szczecin, Wąska 13, 71-712 Szczecin, Poland;

**Correspondence e-mail:** [*anna.cieplok@us.edu.pl*](mailto:anna.cieplok@us.edu.pl)*,*

*corresponding author

e-mail address: [anna.cieplok@us.edu.pl](mailto:anna.cieplok@us.edu.pl),

Faculty of Natural Sciences,

Institute of Biology, Biotechnology and Environmental Protection,

University of Silesia,

Bankowa 9, 40-007 Katowice

Poland

**Table S1**

Raw data on aquatic invertebrates in studied small forest lakes 1 and 2.

|  |  |  | Lake 2 | | | | Lake 2 | | | |
| --- | --- | --- | --- | --- | --- | --- | --- | --- | --- | --- |
| Taxa |  |  | Spring | Summer | Autumn | Mean density | Spring | Summer | Autumn | Mean density |
| Oligochaeta |  |  | 80 | 84 | 36 | 67 | 16 | 12 | 24 | 17 |
| Crustacea | Asellidae |  | 0 | 0 | 0 | 0 | 4 | 0 | 4 | 3 |
| Nematoda |  |  |  | 0 | 0 | 0 | 0 | 0 | 0 | 0 |
| Hirudinea | *Helobdella stagnalis* |  | 4 | 40 | 8 | 17 | 0 | 4 | 0 | 1 |
|  | *Hemiclepsis marginata* |  | 0 | 28 | 0 | 9 | 0 | 0 | 0 | 0 |
|  | *Glossiphonia heteroclita* |  | 0 | 0 | 0 | 0 | 0 | 0 | 0 | 0 |
|  | *Glossiphonia complanata* |  | 0 | 0 | 0 | 0 | 0 | 0 | 0 | 0 |
|  | *Erpobdella nigricolis* |  | 0 | 328 | 0 | 109 | 0 | 0 | 0 | 0 |
|  | *Erpobdella octoculata* |  | 0 | 0 | 0 | 0 | 0 | 0 | 0 | 0 |
|  | *Haemopis sanguisuga* |  | 0 | 0 | 0 | 0 | 0 | 0 | 0 | 0 |
|  | Odonata | Coenagrionidae | 0 | 0 | 0 | 0 | 24 | 0 | 0 | 8 |
|  |  | Lestidae | 12 | 16 | 4 | 11 | 0 | 28 | 4 | 11 |
|  |  | Platycnemidae | 0 | 0 | 0 | 0 | 16 | 0 | 0 | 5 |
|  |  | Libellulidae | 8 | 4 | 0 | 4 | 0 | 0 | 0 | 0 |
|  |  | Cordulidae | 0 | 0 | 0 | 0 | 0 | 4 | 0 | 1 |
|  | Ephemeroptera | Betidae | 0 | 0 | 0 | 0 | 0 | 0 | 48 | 16 |
|  |  | Siphlonuridae | 0 | 0 | 0 | 0 | 0 | 0 | 0 | 0 |
|  |  | Cenidae | 4 | 28 | 20 | 17 | 20 | 0 | 0 | 7 |
|  |  | Leptoceridae | 12 | 12 | 4 | 9 | 24 | 4 | 16 | 15 |
| Insecta | Trichoptera | Ecknomidae | 0 | 4 | 8 | 4 | 0 | 0 | 64 | 21 |
|  |  | Polycentropodidae | 0 | 0 | 0 | 0 | 0 | 0 | 0 | 0 |
|  |  | Phryganeidae | 0 | 0 | 0 | 0 | 0 | 0 | 0 | 0 |
|  |  | Hydropsychidae | 4 | 0 | 0 | 1 |  |  |  |  |
|  |  | Hydroptilidae | 0 | 0 | 0 | 0 | 0 | 4 | 0 | 1 |
|  |  | Chironomidae | 688 | 968 | 1246 | 967 | 1164 | 212 | 432 | 603 |
|  |  | Tabanidae | 8 | 8 | 8 | 8 | 0 | 4 | 0 | 1 |
|  | Diptera | Ceratopogonidae | 8 | 0 | 0 | 3 | 8 | 4 | 0 | 4 |
|  |  | Dixidae | 0 | 0 | 0 | 0 | 0 | 0 | 0 | 0 |
|  |  | Limonidae | 0 | 0 | 4 | 1 | 4 | 0 | 0 | 1 |
|  |  | Haoboridae | 0 | 0 | 0 | 0 | 0 | 0 | 4 | 1 |
|  |  | Haliplidae | 0 | 0 | 0 | 0 | 0 | 0 | 0 | 0 |
|  | Coleoptera | Hydrophilidae | 0 | 0 | 0 | 0 | 0 | 0 | 0 | 0 |
|  |  | Dyiscidae | 0 | 0 | 0 | 0 | 4 | 0 | 4 | 3 |
|  |  | Elmidae | 0 | 0 | 0 | 0 | 0 | 4 | 0 | 1 |
|  |  | Helodidae | 4 | 0 | 0 | 1 |  |  |  |  |
|  |  | Donacidae | 0 | 0 | 0 | 0 | 4 | 0 | 0 | 1 |
|  | Heteroptera | *Ilyocoris cimicoides* | 0 | 0 | 0 | 0 | 0 | 0 | 0 | 0 |
|  |  | *Nepa cinerea* | 0 | 0 | 0 | 0 | 0 | 0 | 0 | 0 |
|  |  | *Plea minutissima* | 0 | 0 | 0 | 0 | 0 | 0 | 0 | 0 |
|  |  | Corixidae | 0 | 0 | 0 | 0 | 0 | 0 | 0 | 0 |
|  |  | *Mesovelia furcata* | 0 | 0 | 0 | 0 | 0 | 4 | 0 | 1 |
|  |  | *Ranatra linearis* | 0 | 0 | 0 | 0 | 0 | 0 | 4 | 1 |
|  | Megaloptera | *Sialis lutaria* | 0 | 0 | 0 | 0 | 0 | 0 | 0 | 0 |
|  | Neuroptera | Planipenia | 0 | 0 | 0 | 0 | 0 | 0 | 0 | 0 |
|  | *Stagnicola palustris* |  | 0 | 0 | 0 | 0 | 4 | 0 | 0 | 1 |
|  | *Anisus spirorbis* |  | 0 | 0 | 0 | 0 | 0 | 0 | 0 | 0 |
|  | *Anisus vortex* |  | 0 | 12 | 0 | 4 | 0 | 12 | 0 | 4 |
|  | *Lymnaea stagnalis* |  | 0 |  | 0 | 0 | 0 | 0 | 0 | 0 |
| Gastropoda | *Gyraulus albus* |  | 0 |  | 0 | 0 | 12 | 12 | 8 | 11 |
|  | *Gyraulus crista* |  | 0 |  | 0 | 0 | 0 | 0 | 0 | 0 |
|  | *Hippeutis complanatus* |  | 0 |  | 0 | 0 | 0 | 16 | 8 | 8 |
|  | *Segmentina nitida* |  | 0 |  | 0 | 0 | 0 | 0 | 0 | 0 |
|  | *Radix balthica* |  | 0 |  | 0 | 0 | 8 | 4 | 0 | 4 |
|  | *Ferrissia fragilis* |  | 0 |  | 0 | 0 | 0 | 4 | 0 | 1 |
|  | *Planorbarius corneus* |  | 0 |  | 0 | 0 | 0 | 0 | 0 | 0 |
|  | *Acroloxus lacustris* |  | 0 |  | 0 | 0 | 0 | 0 | 0 | 0 |
|  | *Viviparus contectus* |  | 0 |  | 0 | 0 | 0 | 0 | 0 | 0 |
| Bivalvia | *Pisidium casertanum* |  | 0 |  | 0 | 0 | 0 | 0 | 0 | 0 |
|  | *Musculium lacustre* |  | 0 |  | 0 | 0 | 0 | 0 | 0 | 0 |
|  | *Sphaerium corneum* |  | 0 | 8 | 0 | 3 | 0 | 0 | 0 | 0 |
| Density in total | |  | 832 | 1540 | 1338 |  | 1312 | 332 | 620 |  |
| Average density per lake | |  | 1237 |  |  | 3710 | 755 |  |  | **2264** |
| Number of taxa | |  | 11 | 13 | 9 |  | 14 | 16 | 12 |  |

**Table S2**

Raw data on aquatic invertebrates in studied small forest lakes 3, 4 and 5.

|  |  |  | Lake3 | | | | Lake 4 | | |  | Lake 5 | | | |
| --- | --- | --- | --- | --- | --- | --- | --- | --- | --- | --- | --- | --- | --- | --- |
| Taxa |  |  | Spring | Summer | Autumn | Mean density | Spring | Summer | Autumn | Mean density | Spring | Summer | Autumn | Mean density |
| Oligochaeta |  |  | 16 | 8 | 8 | 11 | 52 | 316 | 1296 | 555 | 141 | 12 | 852 | 335 |
| Crustacea | Asellidae |  | 0 | 0 | 0 | 0 | 8 | 0 | 8 | 5 | 5 | 0 | 0 | 2 |
| Nematoda |  |  | 0 | 0 | 0 | 0 | 0 | 12 | 12 | 8 | 2 | 0 | 0 | 1 |
| Hirudinea | *Helobdella stagnalis* |  | 0 | 0 | 0 | 0 | 60 | 48 | 28 | 45 | 16 | 0 | 0 | 5 |
|  | *Hemiclepsis marginata* |  | 0 | 0 | 0 | 0 | 8 | 12 | 4 | 8 | 1 | 0 | 0 | 1 |
|  | *Glossiphonia heteroclita* |  | 0 | 4 | 0 | 1 | 4 | 12 | 0 | 5 | 0 | 0 | 0 | 0 |
|  | *Glossiphonia complanata* |  | 0 | 0 | 0 | 0 | 4 | 0 | 0 | 1 | 0 | 0 | 0 | 0 |
|  | *Erpobdella nigricolis* |  | 0 | 0 | 0 | 0 | 12 | 4 | 0 | 5 | 0 | 0 | 0 | 0 |
|  | *Erpobdella octoculata* |  | 0 | 0 | 0 | 0 | 0 | 0 | 4 | 1 | 0 | 0 | 0 | 0 |
|  | *Haemopis sanguisuga* |  | 0 | 0 | 0 | 0 | 0 | 0 | 8 | 3 | 0 | 0 | 0 | 0 |
|  | Odonata | Coenagrionidae | 0 | 0 | 0 | 1 | 12 | 52 | 56 | 40 | 0 | 1 | 88 | 30 |
|  |  | Lestidae | 0 | 28 | 12 | 13 | 24 | 20 | 0 | 15 | 5 | 27 | 0 | 11 |
|  |  | Platycnemidae | 0 | 0 | 0 | 0 | 4 | 44 | 60 | 36 | 0 | 0 | 16 | 5 |
|  |  | Libellulidae | 4 | 0 | 0 | 1 | 0 | 24 | 0 | 8 | 3 | 6 | 0 | 3 |
|  |  | Cordulidae | 0 | 0 | 0 | 0 | 36 | 28 | 0 | 21 | 0 | 0 | 0 | 0 |
|  | Ephemeroptera | Betidae | 0 | 4 | 4 | 3 | 0 | 0 | 552 | 184 | 2 | 2 | 92 | 32 |
|  |  | Siphlonuridae | 0 | 0 | 0 | 0 | 0 | 0 | 0 | 0 | 0 | 4 | 0 | 1 |
|  |  | Cenidae | 4 | 16 | 0 | 7 | 68 | 300 | 604 | 324 | 19 | 62 | 172 | 84 |
|  |  | Leptoceridae | 4 | 0 | 0 | 1 | 4 | 236 | 144 | 128 | 2 | 8 | 26 | 12 |
| Insecta | Trichoptera | Ecknomidae | 0 | 0 | 0 | 0 | 4 | 0 | 0 | 1 | 8 | 12 | 23 | 14 |
|  |  | Polycentropodidae | 0 | 0 | 0 | 0 | 0 | 20 | 24 | 15 | 0 | 0 | 27 | 9 |
|  |  | Phryganeidae | 0 | 0 | 0 | 0 | 0 | 0 | 28 | 9 | 1 | 0 | 14 | 5 |
|  |  | Hydropsychidae |  |  |  |  |  |  |  |  |  |  |  |  |
|  |  | Hydroptilidae | 8 | 0 | 0 | 3 | 0 | 8 | 28 | 12 | 2 | 0 | 2 | 1 |
|  |  | Chironomidae | 364 | 112 | 124 | 200 | 856 | 1436 | 2700 | 1664 | 213 | 103 | 219 | 178 |
|  |  | Tabanidae | 0 | 0 | 0 | 0 | 0 | 4 | 4 | 4 | 0 | 0 | 4 | 1 |
|  | Diptera | Ceratopogonidae | 4 | 4 | 0 | 3 | 16 | 4 | 8 | 9 | 0 | 0 | 4 | 1 |
|  |  | Dixidae | 0 | 0 | 4 | 1 | 0 | 0 | 0 | 0 | 0 | 1 | 0 | 0 |
|  |  | Limonidae | 0 | 0 | 0 | 0 | 0 | 0 | 4 | 1 | 0 | 0 | 0 | 0 |
|  |  | Haoboridae | 0 | 0 | 0 | 0 | 0 | 0 | 0 | 0 | 0 | 0 | 0 | 0 |
|  |  | Haliplidae | 0 | 4 | 0 | 1 | 0 | 0 | 0 | 0 | 0 | 0 | 0 | 0 |
|  | Coleoptera | Hydrophilidae | 4 | 0 | 0 | 1 | 24 | 4 | 0 | 9 | 0 | 1 | 0 | 0 |
|  |  | Dyiscidae | 0 | 44 | 16 | 20 | 0 | 0 | 0 | 0 | 8 | 0 | 4 | 4 |
|  |  | Elmidae | 0 | 0 | 0 | 0 | 0 | 0 | 0 | 0 | 0 | 0 | 0 | 0 |
|  |  | Helodidae |  |  |  |  |  |  |  |  |  |  |  |  |
|  |  | Donacidae | 0 | 0 | 0 | 0 | 0 | 0 | 0 | 0 | 0 | 0 | 0 | 0 |
|  | Heteroptera | *Ilyocoris cimicoides* | 0 | 8 | 0 | 3 | 8 | 8 | 0 | 5 | 5 | 0 | 0 | 2 |
|  |  | *Nepa cinerea* | 0 | 0 | 0 | 4 | 0 | 0 | 0 | 0 | 22 | 0 | 0 | 7 |
|  |  | *Plea minutissima* | 0 | 0 | 0 | 0 | 0 | 0 | 0 | 0 | 1 | 0 | 0 | 0 |
|  |  | Corixidae | 0 | 0 | 12 | 0 | 0 | 16 | 8 | 8 | 0 | 0 | 0 | 0 |
|  |  | *Mesovelia furcata* | 0 | 0 | 0 | 0 | 0 | 0 | 0 | 0 | 0 | 0 | 0 | 0 |
|  |  | *Ranatra linearis* | 0 | 0 | 0 | 0 | 0 | 0 | 0 | 0 | 0 | 0 | 0 | 0 |
|  | Megaloptera | *Sialis lutaria* | 0 | 0 | 4 | 1 | 0 | 8 | 4 | 4 | 0 | 0 | 0 | 0 |
|  | Neuroptera | Planipenia | 0 | 0 | 0 | 0 | 0 | 0 | 4 | 1 | 0 | 0 | 0 | 0 |
|  | *Stagnicola palustris* |  | 0 | 0 | 0 | 0 | 0 | 0 | 0 | 0 | 0 | 0 | 0 | 0 |
|  | *Anisus spirorbis* |  | 0 | 0 | 4 | 0 | 0 | 0 | 0 | 0 | 0 | 0 | 0 | 0 |
|  | *Anisus vortex* |  | 0 | 12 | 4 | 5 | 0 | 0 | 0 | 0 | 0 | 0 | 0 | 0 |
|  | *Lymnaea stagnalis* |  | 0 | 0 | 0 | 0 | 0 | 0 | 0 | 0 | 1 | 0 | 0 | 0 |
| Gastropoda | *Gyraulus albus* |  | 4 | 0 | 4 | 3 | 4 | 56 | 12 | 24 | 7 | 18 | 96 | 40 |
|  | *Gyraulus crista* |  | 0 | 0 | 4 | 1 | 0 | 8 | 0 | 3 | 0 | 0 | 0 | 0 |
|  | *Hippeutis complanatus* |  | 8 | 16 | 16 | 13 | 0 | 40 | 48 | 29 | 0 | 0 | 4 | 1 |
|  | *Segmentina nitida* |  | 4 | 0 | 0 | 1 | 0 | 4 | 0 | 1 | 0 | 0 | 0 | 0 |
|  | *Radix balthica* |  | 0 | 0 | 0 | 0 | 0 | 16 | 12 | 9 | 2 | 0 | 12 | 5 |
|  | *Ferrissia fragilis* |  | 0 | 0 | 12 | 4 | 0 | 16 | 16 | 11 | 0 | 0 | 92 | 31 |
|  | *Planorbarius corneus* |  | 0 | 0 | 0 | 0 | 0 | 0 | 16 | 5 | 2 | 99 | 0 | 34 |
|  | *Acroloxus lacustris* |  | 0 | 0 | 0 | 0 | 0 | 8 | 20 | 9 | 0 | 0 | 4 | 1 |
|  | *Viviparus contectus* |  | 4 | 0 | 0 | 1 | 0 | 0 | 0 | 0 | 0 | 0 | 0 | 0 |
| Bivalvia | *Pisidium casertanum* |  | 0 | 0 | 12 | 4 | 0 | 4 | 4 | 3 | 1 | 0 | 0 | 0 |
|  | *Musculium lacustre* |  | 0 | 0 | 0 | 0 | 4 | 0 | 4 | 3 | 0 | 0 | 0 | 0 |
|  | *Sphaerium corneum* |  | 0 | 0 | 0 | 0 | 4 | 0 | 0 | 1 | 0 | 0 | 0 | 0 |
| Density in total | |  | 432 | 260 | 240 |  | 1216 | 2768 | 5720 |  | 470 | 356 | 1751 |  |
| Average density per lake | |  | 311 |  |  | **932** | 3235 |  |  | **9704** | 859 |  |  | **2577** |
| Number of taxa | |  | 13 | 12 | 15 |  | 21 | 30 | 30 |  | 24 | 14 | 19 |  |

**Table S3**

Data on water chemistry measures in small forest lakes during the study period. Temp.- temperature, Cond.- Conductivity, TDS- total dissolved solids, Alk.-alkalinity.

|  |  | Temp. | pH | Cond | TDS | O2 | Hardn | Alk | Cl | Ca | N- NH4 | NH3 | N-NO3 | NO3 | N-NO2 | NO2 | PO4 | SO4 | Fe | Organic matter |
| --- | --- | --- | --- | --- | --- | --- | --- | --- | --- | --- | --- | --- | --- | --- | --- | --- | --- | --- | --- | --- |
| Lake 1 | Spring | 25 | 6 | 230 | 110 | 10,5 | 110 | 70 | 30 | 40 | 0,91 | 1,11 | 0 | 0 | 0 | 0 | 0,11 | 2,45 | 24 | 21,84 |
|  | Summer | 26 | 7 | 180 | 80 | 7,62 | 140 | 55 | 22 | 32 | 1,02 | 1,24 | 0 | 0 | 0 | 0 | 0,37 | 2,35 | 2,35 | 6,41 |
|  | Autumn | 18 | 8 | 190 | 80 | 9,72 | 140 | 75 | 24 | 62 | 0,51 | 0,62 | 0,7 | 3,1 | 0 | 0 | 0,09 | 2,1 | 1,2 | 7,81 |
| Lake 2 | Spring | 25,8 | 7 | 220 | 100 | 10,7 | 112 | 60 | 28 | 42 | 0,51 | 0,62 | 0 | 0 | 0,005 | 0,02 | 1,22 | 2,48 | 29 | 2,53 |
|  | Summer | 25,6 | 7 | 290 | 130 | 6,96 | 155 | 75 | 20 | 32 | 0,81 | 0,98 | 1 | 4,43 | 0 | 0 | 2,5 | 2,1 | 1,19 | 4,91 |
|  | Autumn | 17,9 | 8 | 180 | 80 | 7,8 | 110 | 80 | 38 | 85 | 0,44 | 0,53 | 0,5 | 2,21 | 0 | 0 | 0 | 1,8 | 0,86 | 2,44 |
| Lake 3 | Spring | 24,4 | 7 | 220 | 100 | 3,36 | 114 | 60 | 28 | 42 | 0,51 | 0,62 | 0 | 0 | 0,005 | 0,02 | 1,22 | 2,48 | 29 | 25,31 |
|  | Summer | 25,8 | 7 | 190 | 90 | 6,2 | 148 | 100 | 24 | 30 | 0,55 | 0,66 | 0 | 0 | 0 | 0 | 0,17 | 2,38 | 1,85 | 34,81 |
|  | Autumn | 18,1 | 7 | 190 | 90 | 8,44 | 140 | 95 | 50 | 70 | 0,46 | 0,56 | 0 | 0 | 0 | 0 | 0,06 | 2,1 | 0,93 | 3,82 |
| Lake 4 | Spring | 24,6 | 7 | 270 | 120 | 6,75 | 125 | 50 | 18 | 36 | 0,85 | 1,03 | 0,1 | 1,9 | 0 | 0 | 0,1 | 30 | 0,63 | 0,65 |
|  | Summer | 25,4 | 7 | 190 | 80 | 7,79 | 150 | 120 | 22 | 32 | 0,78 | 0,94 | 0,1 | 1,9 | 0 | 0 | 1,33 | 28 | 0,43 | 2,74 |
|  | Autumn | 19,1 | 7 | 180 | 80 | 9,06 | 120 | 100 | 38 | 78 | 0,39 | 0,47 | 0,9 | 17,6 | 0,002 | 0,01 | 0 | 31 | 0,31 | 1,12 |
| Lake 5 | Spring | 25 | 7 | 240 | 110 | 7,82 | 110 | 50 | 22 | 30 | 0,67 | 0,81 | 0,6 | 2,65 | 0 | 0 | 0,29 | 29 | 0,3 | 14,04 |
|  | Summer | 25,8 | 7 | 180 | 90 | 8,56 | 152 | 100 | 24 | 30 | 0,7 | 0,85 | 0,7 | 3,1 | 0 | 0 | 0,15 | 28 | 0,08 | 4,54 |
|  | Autumn | 20,8 | 7 | 190 | 80 | 10,5 | 130 | 125 | 32 | 68 | 0,38 | 0,46 | 0,3 | 1,32 | 0 | 0 | 0,28 | 27 | 0,07 | 43,22 |

**Table S4**

Data characterizing the studied forest lakes.

|  |  | Number of lake | | | | |
| --- | --- | --- | --- | --- | --- | --- |
|  |  | 1 | 2 | 3 | 4 | 5 |
| Sampling site | latitude | 50.658104° | 50.657669° | 50.657616° | 50.651901° | 50.646828° |
|  | longitude | 18.476545° | 18.468885° | 18.466591° | 18.453382° | 18.447107° |
| Total area | ha | 3,85 | 7,85 | 7,85 | 8,79 | 13,57 |
| Area of water | ha | 3,67 | 7,46 | 7,26 | 8,33 | 12,98 |
| Ordinate water (overflow) | m n.p.m. | 217 | 216 | 215 | 213 | 211,8 |
| Overflow in the bottom | m n.p.m. | 214,93 | 213,4 | 212,4 | 210 | 207,2 |
| Average lake capacity | m3 | 38940 | 86040 | 90600 | 95760 | 231990 |
| Average depth | m | 1,1 | 1,2 | 1,2 | 1,2 | 1,85 |

**Figure S1.**


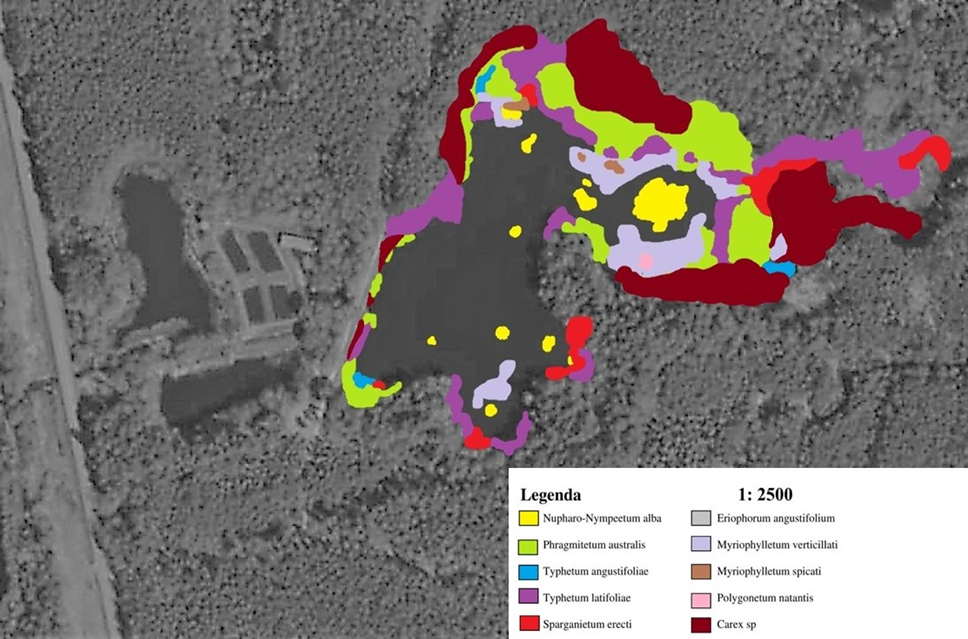
Model of plant associations in the studied artificial forest lake 1.

**Figure S2.**


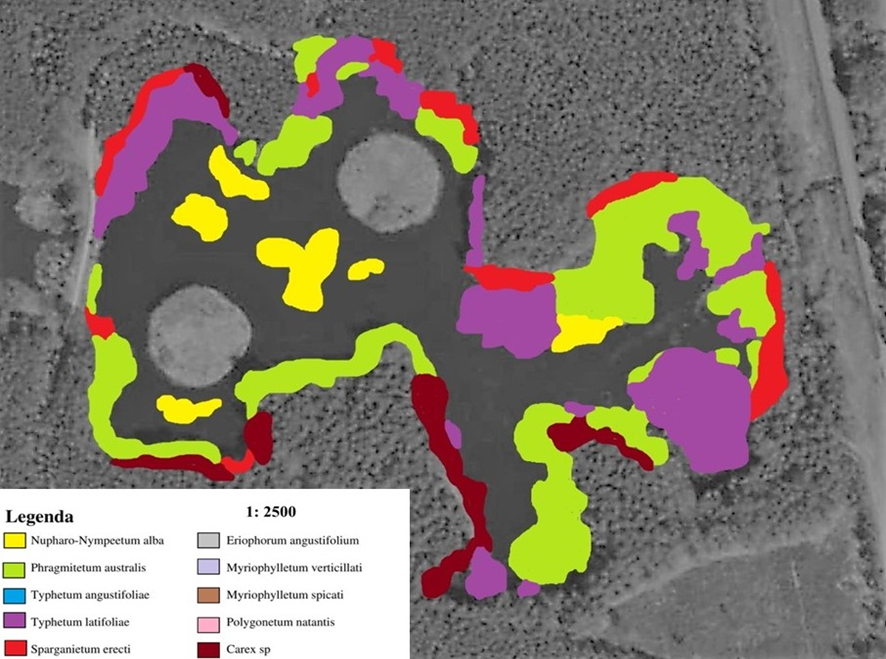
Model of plant associations in the studied artificial forest lake 2.

**Figure S3**

Model of plant associations in the studied artificial forest lake 3.


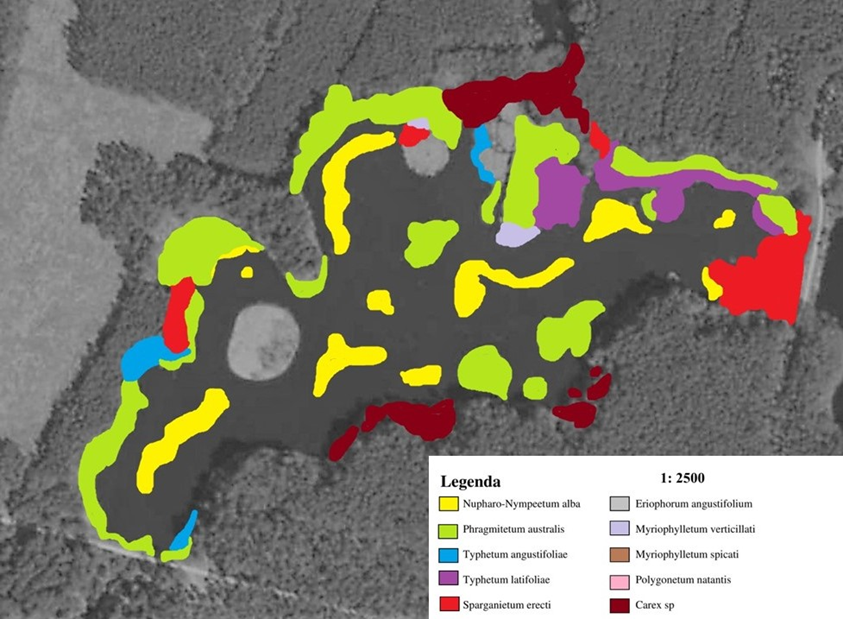


Rycina 3. Roślinność rzeczywista stawu nr 3

**Figure S4**

Model of plant associations in the studied artificial forest lake 4.


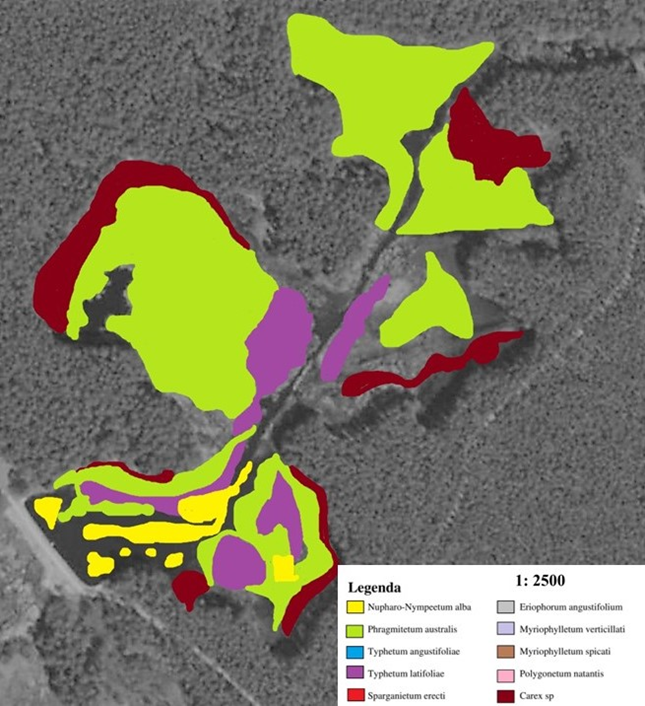


**Figure S5**


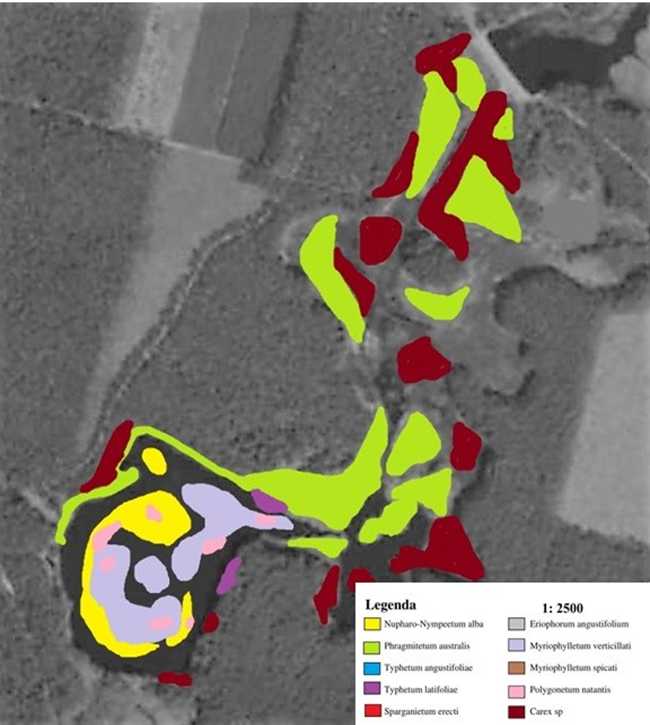
 Model of plant associations in the studied artificial forest lake 5.
